# Securitization versus sovereignty? Multi-level governance, scientific objectivation, and the discourses of the Canadian and American heads of state during the first wave of the COVID-19 pandemic

**DOI:** 10.1177/00207020241275980

**Published:** 2024-08-28

**Authors:** Marjolaine Lamontagne

**Affiliations:** Department of Political Science, 5620 McGill University, Montréal, Québec, Canada

**Keywords:** COVID-19, federalism, multi-level governance, politicization, sovereignty, securitization, Canada, United States, global health governance

## Abstract

The global health regime is caught in a paradox, whereby connecting “human” to “(inter)national” security to prevent the spread of infectious diseases unwittingly introduces into this complex and expertise-reliant domain of “low politics” the notion of “sovereign decisionism”—states’ prerogative to identify a threat and counter it with exceptional measures that may in turn constrain their ability to unilaterally securitize disease. This article introduces an analytical framework presenting three pathways through which state leaders with different conceptions of sovereignty and varying constraints on their legitimacy among their domestic audiences may nevertheless securitize policy domains traditionally considered as falling within the scope of sub-state “low politics.” Two of the pathways begin with scientific objectivation rather than politicization, and one trades power concentration for collaboration with sub-state and global authorities. I then compare the Canadian and American responses during the first wave of the coronavirus pandemic to uncover how these contextual factors disposed Donald Trump to *politicize* COVID-19, while Justin Trudeau emulated the World Health Organization's *securitization* of the virus without centralizing state powers.

“I will always put the well-being of America first…. The virus will not have a chance against us. No nation is more prepared or more resilient than the United States.” American president Donald Trump's speech to the nation on 11 March 2020 portrayed the COVID-19 pandemic as a “very dangerous health threat” “from China,” “seeded from European travelers,” and which would be “expeditiously defeated.”^1^ Meanwhile, across the northern border, Canadian prime minister Justin Trudeau was addressing his constituents with a much less divisive and more prudent tone: “the number of people affected by the virus around the globe keeps climbing…. I want all premiers and all Canadians to know: Our government is here for you.”^2^ Both leaders were responding to the World Health Organization's (WHO) announcement that SARS-CoV-2 had become a “global pandemic,” and were equally called upon to ensure their nation's welfare and security in the face of the greatest crisis the international community had faced since the Second World War. Yet, because the pandemic constituted a “non-traditional” security threat, the management of which required the expertise of international and national technocrats and fell largely within the jurisdiction of sub-state governments, Trump and Trudeau were constrained in their ability to take decisive action against the virus on their own.

National security is traditionally the prerogative of central states and a core foundation of state sovereignty. According to Ole Waever's “classical” securitization theory (CST), when leaders designate an issue a “security” matter (i.e., when they “speak security”), they introduce and legitimize in that sector of activity “sovereign decisionism”—that is, the sovereign's absolute prerogative to “decide whether there is to be an extreme emergency as well as what must be done to eliminate it.”^3^ In this way they reinforce their own authority vis-à-vis other actors at the international and sub-state levels by demanding support for the enactment of “exceptional” measures that override the rules of normal politics. The global health regime, which has sought to securitize pandemic influenzas since the 1990s,^4^ is therefore caught in a paradox, whereby attempts to compel states into taking preventive action against infectious diseases by linking “human security” (i.e., the protection of individuals’ health and welfare) with *national and international security*,^5^ conflict with the intrinsically “multi-level” and expertise-based nature of healthcare governance by unwittingly encouraging state executives to introduce sovereign decisionism into this policy domain. As noted by the expanding global health literature in International Relations (IR), “[the field's] statist focus is in tension with more cosmopolitan visions of global health, which require broader health system strengthening.”^6^ A productive angle from which to examine this tension is through the lens of security. Recent research on the management of global health threats has indeed shown that states’ “desire and capacity to securitize infectious disease is complex and cannot be assumed.”^7^ In this regard, Canada and the US are institutionally similar cases with highly divergent outcomes: both are developed liberal democracies and highly decentralized federations in which most powers related to healthcare and policing rest with the federated governments, and yet, the difference between Trump's and Trudeau's responses to the pandemic could not be starker. As evinced by this research, the former deployed a discourse that *politicized*, rather than securitized, the pandemic, while the latter securitized it *without* attempting to claim additional authority for the federal state.

This article is interested in uncovering the ideational, institutional, and political contexts underlying state leaders’ propensity to securitize—or, on the contrary, *not* securitize— low politics issues involving the legislative powers of sub-state government and a high degree of technocratic expertise at the national and international levels. Such low politics issues include policy domains like climate change mitigation that are traditionally assumed not to affect or involve the regalian functions of the sovereign state unless they have been subjected to a successful process of securitization.^8^ This research to explore the relationship between securitization and sovereignty, by examining if securitization is always synonymous with attempts by national leaders to concentrate power within the central state, and if states of “exceptionalism” can arise in a context of “multi-level governance” and without prior politicization of the “referent subject” of security (the “threat”). In an era of globalization characterized by the proliferation of non-traditional security “threats” (such as climate change) and increasing diffusion of authority “above,” “alongside,” and “under” the nation state, understanding this relationship is essential, especially in liberal democratic contexts where power concentration is neither an institutional given nor a desirable outcome. To do so, the research brings together two literatures that are seldom in conversation: securitization studies and multi-level governance and federalism.

In the former literature, several scholars have begun addressing the multi-sectoral, multi-scalar, and multi-level dimensions of security,^9^ but more work is needed to understand how securitizing actors at the state level interact with specific domestic audience constraints such as the formal *sharing* and *division* of *sovereignty—*a founding principle of federal regimes that goes beyond the mere “diffusion” of authority—and the public acceptance of technocratic assessments of “threats,” and how this may influence their propensity to securitize low politics. In other words, what can the potential securitization of decentralized, scientifically objectivized policy domains signify for prevailing understandings of state sovereignty as “indivisible” in IR? Multi-level governance is a feature of any modern state and is notably a consequence of the expansion of global and supranational governance in the past three decades. However, federal regimes pose a particular challenge to conceptions of sovereignty that emphasize the concentration and centralization of rule, because they are built on the principle of a “pact” between constituent entities with legislative and executive powers that are supported by historical institutions with considerable political and symbolic weight.^10^ For this reason, federations constitute critical cases through which we can begin re-examining domestic political processes—such as securitization, partisan polarization, and the politicization of international organizations and global authority—that have begun attracting the attention of IR scholars due to their growing and significant impacts on global governance and international security.

Conversely, while students of federalism and multi-level governance have produced a rich body of work demonstrating the influence of power decentralization, federal institutions, and politicization on states’ response to the pandemic,^11^ none have focused on the explicit links between these factors and the *securitization* of COVID-19. To put it differently, while they have carefully examined whether the pandemic affected (de)centralization and intergovernmental cooperation dynamics in federations,^12^ they have not considered the broader implications of beginning to view healthcare primarily as a *security matter* for the conduct of multi-level governance and federal relations in the twenty-first century. The purpose of this research is to open a critical discussion on this topic.

To this end, this article draws together recent theoretical amendments to CST and literatures on global and multi-level governance to present an original analytical framework positing three alternative “pathways” through which national leaders may attempt to justify and implement a state of exception. In contrast to the standard account of CST, only two of these pathways involve efforts by national leaders to centralize decision-making powers at the central state level, and two out of three are dependent on a technocratic, instead of a political, assessment of “threat.” My central argument is that sovereign state leaders can be viewed as “pragmatic agents”^13^ whose propensity to securitize a low politics issue and the way they may do so (i.e., the pathway taken, which may or not involve politicization and/or power concentration) is in great part (though not exclusively)^14^ shaped by their varying understandings of what “sovereignty” entails and the *legitimacy constraints* imposed on them by relevant national audiences—namely, the citizens and sub-state leaders whose compliance is required to institute a nation-wide state of exceptionalism.

My comparative analysis of the public discourses and public health decisions of the Canadian and American heads of state allowed me to identify two sources of constraints that appear to be particularly salient in the securitization of low politics, namely: 1) the multi-level diffusion of authority, which is underpinned by competing conceptions of how power ought to be distributed, especially in federations; and 2) domestic levels of affective (i.e., partisan) polarization surrounding the politicization of global authority and scientific expertise, particularly within the central state party system. After explaining how CST may be amended to better account for the (potential) securitization of low politics issues, I showcase the analytical utility of my proposed framework through the analysis of Canada and the US. I demonstrate that, depending on leaders’ conception of state power and how it may be pragmatically enacted with regard to audience-related constraints, securitization is not inevitable, is not always tantamount to increased “sovereignty” (understood in the narrow sense of power concentration), and may indeed take highly decentralized and expertise-reliant low politics issues as its referent subject.

## Amending classical securitization theory: Securitizing actors as pragmatic agents

Historically, the claim to protect a national population against internal and external threats has been the main foundation of state sovereignty. After the Cold War ended, there emerged a “new security agenda” extending to low politics matters that used to be of little concern to sovereign agents, prompting authors such as Ole Waever and Barry Buzan to reconceptualize security in terms of its “logic” rather than its focus on territorial defense, war-making, and the military sector.^15^ They consequently define securitization as the process of staging diverse referent subjects “as existential threats to a referent object by a securitizing actor who thereby generates endorsement for emergency measures beyond rules that would otherwise bind.”^16^

Securitization can therefore be conceived as an extreme form of politicization, whereby an issue that was previously made into a matter of public debate by elites is successfully objectified, that is, framed as sufficiently urgent to suspend further political deliberation and justify the concentration of executive power in the hands of a select number of securitizing actors, typically heads of state. Although ST scholars highlight the increasing number of non-state actors currently involved in the definition and enactment of national and international security,^17^ the association between security, securitization processes, and state sovereignty remains strong, as “sovereign decisionism” is progressively introduced into a growing number of policy sectors, from international migration to climate change.^18^

Securitization theorists indicate how “threats are ‘objective’ when they are accepted by significant political actors, not because they have an inherent threatening status.”^19^ In this respect, securitization theory diverges from the crisis management literature, which has produced numerous studies on state management of COVID-19.^20^ This literature examines the various societal, psychological, and political factors that influenced leaders and experts’ responses to understand why some were more successful than others in interpreting and managing what it assumes to be an *objective* threat. However, in building off this assumption, most extant research sidesteps more fundamental questions regarding the potential for such episodes of crisis to transform authority relationships—between global, national, and sub-state public authorities, as well as between civil societies, governments, and scientific and technocratic bodies— which have slowly become institutionalized and naturalized since the modern era, and which are deeply anchored in political struggles of meaning-making and legitimacy. Conversely, by looking at threats as socially constituted phenomena, securitization theory provides a better angle from which to discuss sovereignty, while providing us with tools to interpret leaders’ responses to catastrophes. Indeed, even a virus that can endanger the health and lives of millions of people from a biological standpoint is only a “threat” in a *social *sense if the victims' lives are considered valuable enough to be prioritized over other considerations, such as the preservation of established economic and political orders. 

With this interpretive goal in mind, I also retain from the crisis management literature the crucial insight that the first stages of a perceived “crisis” constitute a critical juncture in which high uncertainty regarding the unfolding of events generate conflicting evaluations of a situation.^21^ Such interpretations, I contend, are underpinned, where heads of state are concerned, by competing understandings and enactments of state sovereignty that may be less visible in normal times. This “sense-making” stage of interpretation is followed by a “decision-making” stage, both of which are heavily influenced for leaders by the anticipated reaction of their “audience.”^22^

The concept of “audience” is perhaps the most elusive and undertheorized dimension of ST,^23^ as it problematizes exactly whom must accept securitization for exceptional measures to be successfully enacted, and which audience characteristics may facilitate^24^ or inhibit securitization. Thierry Balzacq contributes a crucial amendment to CST by contending that securitization is an agonistic, intersubjective process that requires the assent of relevant audiences—in other words, those whose support is needed to enact exceptionalism—and which occurs in a field of power. Securitizing actors, in other words, are *pragmatic* political agents who must consider the sociopolitical context in which they operate to successfully elevate an issue above normal politics and generate support for a state of emergency.^25^ This requires a continuous evaluation of what this study refers to as their *legitimacy constraints*, that is, their degree of legitimacy in the eyes of their audience. In other words, they must have a clear grasp of the position from which they are speaking and to what extent it allows them to credibly claim knowledge about the referent subject, as well as the authority to manage the threat it presents with regard to their audiences’ collectively held ideas.

Political leaders are invested in the diffusion and construction of audiences’ intersubjective realities, even as their own identities and interests are shaped by these same social constructs.^26^ Therefore, they tend to position themselves in the political field in order to exercise power in ways that align with the meanings they ascribe to the state's *internal* and *external sovereignty*, which correspond to their various understandings of how state power ought to manifest, and how authority should be distributed or shared within the state or with other technocratic or supranational actors.^27^ Conceptions of sovereignty hence inform leaders’ various dispositions regarding the discursive and political enactment of sovereignty, given their position in the agonistic field of intersubjective ideas held by themselves and relevant audiences. Following the cue of crisis management literature, the present analysis posits that the relationship between sovereignty and low politics securitization can best be captured by focusing on the first wave of the pandemic, that is, when the “critical juncture” thus created offered heads of state an opportunity to promote particular conceptions of sovereignty through illocutionary speech acts, or “doing by saying.” These speech acts, in turn, could produce policy consequences if accepted by relevant audiences, a process ST defines as perlocution,^28^ hence potentially opening future avenues for path-dependent centralization *or* diffusion of executive powers. In the following two sections, I identify two main *legitimacy constraints* which directly shape state leaders’ dispositions when it comes to the (potential) securitization of decentralized and expertise-reliant low politics issues like COVID-19: first, the multi-level diffusion of authority, and second, the degree of domestic affective (partisan) polarization surrounding the politicization of national and global scientific authority.

### Legitimacy constraint 1: Multi-level governance

The ability of heads of states to convincingly present themselves as purveyors of security in non-traditional sectors may be severely complicated by the diffusion of governance inherent to many “low politics” matters. Liesbet Hooghe and Gary Marks have argued that under globalization, “modern governance is dispersed across multiple centers of authority.”^29^ They distinguish two types of multi-level governance (MLG). Type I MLG arrangements are characterized by a limited number of general-purpose, territorialized, non-intersecting jurisdictions bundling together multiple functions, policy responsibilities, “and, in many cases, a court system and representative institutions.”^30^ Federal systems are the most advanced form of type I MLGs, as they implement a constitutional division and sharing of powers across two or more levels of government, a regime type in which, according to Daniel Elazar, the traditional understanding of sovereignty as unified control is modified to allow for “[d]ifferent governments [to] relate to one another as *equals* with regard to the powers delegated to each respectively.”^31^ In real-life federal states, competence encroachments between levels of government frequently occur, as actors holding conflicting views about the proper practice of federalism claim authority over matters not part of their jurisdiction. Political parties in federations can also diverge on their views of how centralized the federation ought be (i.e., what is the appropriate level of autonomy for federated entities), as well as on their perception of how horizontal, hierarchical, cooperative, or dual intergovernmental relations should be.^32^ In both the US and Canada, we find important differences in visions of federalism among the main ruling parties, as well as among federated party leaders and heads of state. These, in turn, have a demonstrated influence on policy-making at all levels of government,^33^ including in times of crisis. Many federal scholars like Yvonne Hegele and Johanna Schnabel^34^ have focused on the influence of institutional variables to explain differing patterns of intergovernmental cooperation and (de)centralization among liberal federations during the pandemic; however, they have mostly neglected the influence of ideational factors, critical among which are prevalent and competing understandings of federalism among political elites and the broader electorate. In doing so, they have missed an opportunity to highlight the significance of witnessing, in many of these federal cases, the emergence of exceptionalism alongside a surprisingly low prevalence of intergovernmental conflict and the remarkable upholding of federated autonomy in the midst of an acute “security” crisis.

Indeed, the historical purpose of federal states is to monopolize the external use of force and manage national security *on behalf* of their constituent units.^35^ What then, are the potential implications of securitizing issues belonging to the jurisdiction of sub-state governments for the distribution of power in federal states? Do episodes of crisis create an opening for sovereign decisionism to encroach upon sub-state autonomy? In theory, securitization of low politics matters should incentivize federal state leaders to invoke national security as a means to re-centralize sub-state powers; however, constitutionally entrenched decentralization can present a significant obstacle to securitization attempts,^36^ hence discouraging leaders from trying it in the first place. As the analysis of the Canadian and American cases will show, the relationship between security and sovereignty is much more complex in practice, evincing the trade-offs federal leaders make as pragmatic agents: Trump championed a very populist and majoritarian understanding of sovereignty throughout his mandate as president, and, unsurprisingly, validated the second expectation by showing reluctance to securitize a health emergency over which American states held considerable authority. Trudeau, however, defied both theoretical expectations outlined above by deploying a securitization discourse that respected provincial jurisdiction, in large part thanks to a much more liberal and flexible interpretation of what “sovereign authority” entails. In contrast to MLG type I, type II MLG “is fragmented into functionally specific pieces” and “specialized jurisdictions,”^37^ and usually involves diffusion of state authority “sideways” to public and transnational agencies and “upwards” to international and supranational institutions in functional domains like trade and global health, generating other types of constraints for sovereign executives, as discussed below.

### Legitimacy constraint 2: Domestic polarization and the politicization of global authority

The literature on global health shows that infectious diseases have been securitized by national and international agencies since the 1990s, with securitization becoming firmly established with the expansion of the WHO's powers under the revised International Health Regulations of 2005 and the publication of the 2007 World Health Report, “A Safer Future: Global Public Health Security.”^38^ Despite the ethical dilemmas involved in linking health with security, the connection was progressively made to encourage states to devote greater resources to the prevention and management of epidemics.^39^ More broadly, the WHO's securitization of infectious diseases reflects the progression of a “human security” paradigm that seeks to construe viruses as the referent subjects of security and the integrity and well-being of individuals (rather than states) as the main referent objects. The paradigm has so far been adopted by the UN, the EU, and several countries such as Canada, Japan, and Norway.^40^ This trend, in turn, testifies to the post-1990 evolution of an “international system” dominated by sovereign states towards a “global order” in which transnational bodies—whether IGOs, transnational agencies, or the EU—exercise authority “across national borders” and seek to subordinate sovereign authority to the pursuit of a global “common good” and the protection of human rights.^41^ The global health regime is buttressed by a transnational technocracy incorporating international expertise and national public health agencies (such as the Center for Disease Control [CDC] and the Public Health Agency of Canada) which are partly subject to the coordination efforts of the WHO. This technocracy's dominant discourse generally maintains that the globalization of infectious disease should be met with global solutions but has struggled to impose its norms of disease management and control on states in previous epidemics.^42^

Far from vitiating sovereign independence, however, the rising authority of international institutions has caused them to become increasingly politicized and contested by leaders and civil society, a process that has been shown by Catherine De Vries, Sarah Hobalt, and Stephanie Walter to be heavily mediated by domestic politics, partisan systems, and institutions.^43^ First, there exists a complicated relationship between the main political ideologies articulated within a country's party system and the susceptibility of politicians and the wider public to defer to global authorities and scientific expertise. As the past decade of research has shown, political parties in Western democracies are repositioning themselves along a new “transnational cleavage” (also known as “GAL/TAN”), which redistributes political affiliations, inter alia, in terms of relative support towards globalization, international cooperation, and the legitimacy of international institutions.^44^ Second, the politicization of international cooperation and institutions can be connected to dynamics of “affective polarization”—the development of partisan sorting and cross-partisan hostility—within states, as is increasingly the case in the US.^45^ When politicization and polarization are tightly entwined, opposition to international institutions and skepticism towards scientific research can become the objects of intense political competition,^46^ which can in turn hinder processes of scientific objectivation or the technocratic securitization of low politics.

“Technocratic securitization,” a term coined by Anders Esmark to designate a *scientific*—rather than a *political*—identification of health threats, refers to a mode of governance that relies on highly specialized expertise to curtail political debate by imposing a single interpretation of complex events or crisis episodes through authoritative claims of scientific knowledge.^47^ A related concept brought forth by Trine Villumsen Berling, *scientific objectivation*, describes the process of appealing to scientific knowledge to put an end to political and social debates. Whereas the scientific field is often traversed by vigorous disagreements and high levels of uncertainty, it is depicted from the outside as a unified field of knowledge meant to deliver *complete* and *objective* information to other sectors of society that can effectively stifle political disagreement.^48^ When COVID-19 emerged, the transnational technocracy underpinning the global health regime deployed a “securitization grammar of defense against existential threats and all-out fight against an enemy at the door,”^49^ ultimately compelling the majority of states to declare a state of emergency when death tolls began to rise.^50^ Public health responses around the globe were thus justified by invoking a technocratic principle of *precaution*, which demanded intrusive measures “harking back to the oldest of sovereign prerogatives” and amounted to a direct infringement on civil liberties (via such measures as quarantines, border closures, surveillance, and mask mandates) in order to prevent *future*, *potential* harm.^51^ In other words, leaders who adopted (or co-opted) the WHO's securitization discourse regarding COVID-19 accepted that important socioeconomic sacrifices ought to be made in the short term to prevent even greater damages to societies, national economies, and international security in the long run. In essence, it was acknowledged that national and international security depended on temporarily prioritizing citizens’ lives and health over *other* valued referent objects, such as individual liberties and economic welfare.^52^

**Figure 1.**
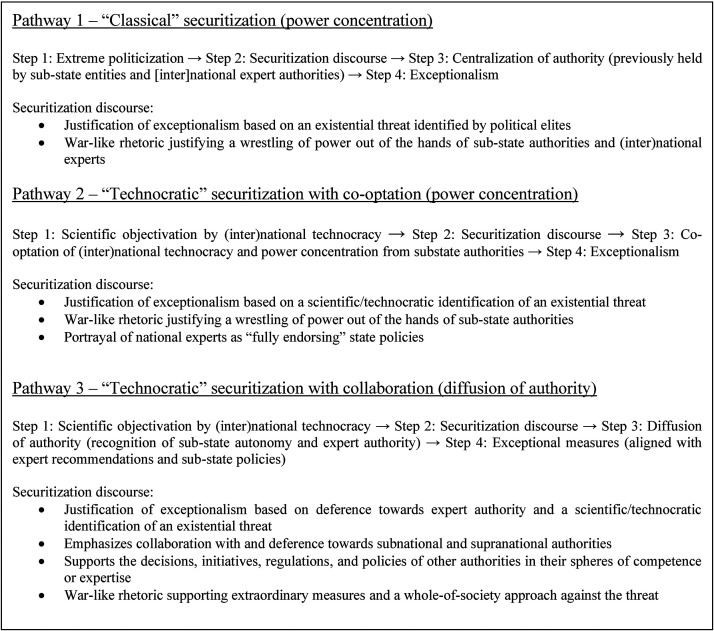
Three alternative pathways to exceptionalism and related variations in securitization discourse.

In summary, the technocratic management of crises and the scientific objectivation of threats tend to produce a dual effect, therefore modifying the traditional pathway to exceptionalism depicted in CST. First, they neutralize the politicization of subject referents (i.e., they make contestation and debate difficult or impossible. Second, they serve as justification for the exercise of power by the *relevant* authorities, introducing the necessary conditions for the swift adoption of exceptional measures.^53^ Concerning the concentration of powers, two scenarios are possible. In the first, central state executives can *defer* to the expertise of technocratic elites and delegate a substantial part of their authority in crisis management, which was the chosen course of action for many states including Germany and Sweden. Acknowledgement of power decentralization and strategies of “cooperative federalism” also constitute a form of deference to another level of authority, this time directed towards sub-state governments. The other scenario involves the co-optation of national expertise by sovereign executives, who thereby invoke technocratic discourses *without* deferring authority. This was the preferred strategy of many populist leaders during the pandemic. In both scenarios (diffusion/collaboration and co-optation), the paradoxical result is securitization *without* prior politicization and a state of emergency that is mainly justified through the invocation of a technocratic/scientific “consensus.” This alternative pathway to exceptionalism, which may or may not be conducive to greater sovereign decisionism, differs from the traditional pathway from securitization to exceptionalism presented by CST, which begins with politicization and ends with increased sovereignty. All three pathways are presented in Figure 1 below.

Finally, there is the possibility that exceptionalism itself may be rejected or undermined by state elites—for instance, in cases when the technocratic consensus about the existence of a threat is deliberately ignored or denied. The literature on the effects of populism on pandemic management yielded surprising results: according to Anders Esmark, populism as a “style of politics…which invokes radical concepts of popular sovereignty in the assertion of a monopoly on representation of ‘the people’ against its enemies,” cannot account on its own for the (un)willingness of heads of state to securitize an issue—one must also consider the particular ideology to which a leader subscribes.^54^ Contrary to many other populist leaders around the globe, Trump engaged in crisis denial and displacement in lieu of instrumentalizing the pandemic to stage an autocratic bid to power. In doing so, he validated the two theoretical expectations that, as outlined above, would normally flow from a traditional conceptualization of sovereignty as *indivisible* rule, by refusing to securitize an issue that would have required the head of state to defer to international technocratic authority and to the legislative powers of sub-state governments. In comparison, the Canadian case evinces how *differing conceptions* of sovereignty, perhaps better aligned with the principles of federalism and the evolution of state/non-state, domestic/international relations in the twenty-first century, can conversely lead to the securitization of low politics and the emergence of exceptionalism amidst a web of collaborative authority relationships.

### Methodology

Based on the analytical framework presented in above, I retained three criteria to evaluate whether the discourses of federal leaders could qualify as “securitizing” COVID-19, and then used those criteria to hand-code a delimited corpus of Trudeau's and Trump's public speech acts. The criteria are as follows:

1. *The virus, or pandemic, itself is consistently framed as the* main *referent subject of security*, although potential vectors of the disease may also be securitized (e.g., particular behaviours, like social gatherings, or groups of people, like non-citizens and migrants).
2. *The discourse follows the typical script of securitization*, whereas the virus as subject referent is consistently presented as an *existential “*threat” to valued referent objects, to justify the adoption of exceptional public health measures. This framing, depending on the case, may or may not align with the WHO's own narrative linking human to national and international security.
3. “*War-like” metaphors are frequently employed*: many scholars have associated the regular use of such metaphors as a tell-tale indicator of securitization,^55^ and others have further drawn a distinction between a rhetoric of “risk” and a rhetoric of “threat.” The former emphasizes “future uncertainty” as opposed to “immediate” danger, as well as the need for a long-term “perpetual management of vulnerability” (danger as arising from “conditional causes”) as opposed to a rapid and *temporary* mobilization to eliminate a clear and unconditional threat.^56^

Consistency of discourse is highlighted in all three criteria because it constitutes a basic condition for the successful elevation of the subject of securitization “above” the normal politics of democratic deliberation. Consistency in the framing of the pandemic can either denote a process of a) extreme politicization (i.e., the threat is continuously magnified by leaders); b) scientific objectivation (i.e., deference to non-state authorities’ assessment of the threat); or c) a combination of the above (i.e., co-optation of expertise). Inconsistency, for its part, may betray an *unwillingness* or *hesitancy* to securitize. I retained a periodization broadly corresponding to the first wave of the pandemic in North America, extending from January to July 2020.^57^ The analysis relies on secondary sources on the political management of the pandemic in both countries, and on a corpus of primary mediatic sources. The Canadian corpus is composed of the verbatims of the eighty-one televised addresses to the nation pronounced by Trudeau from 11 March 2020 to 1 July 2020. The US corpus consists of the verbatim of Trump's 11 March address to the nation and of detailed compilations of his Twitter feed found in media articles and the secondary literature.

## “Team Canada.” Justin Trudeau's technocratic and collaborative securitization of COVID-19

When the COVID-19 pandemic first reached Canada on 25 January 2020, Prime Minister Trudeau was managing a serious conflict between Indigenous communities in Canada and the federal government, which might explain the slowness of his initial response. Nevertheless, in deciding against enacting travel bans on countries affected by the virus and in choosing to keep the southern border open, Canadian minister of health Patty Hajdu was broadly in line with WHO recommendations concerning the continuation of international travel, although screening, testing, and quarantine procedures at the border and in airports were implemented belatedly and fell well below the standards established by the IGO.^58^

However, things changed drastically between 11 March and 18 March, the latter the date when the US border was officially closed to non-essential travelers due to mounting pressure from the provinces (the city of Montreal even sent policy officers to Pierre-Elliott-Trudeau International Airport to distribute pamphlets on quarantine guidelines to travelers).^59^ Beginning on 11 March, Prime Minister Trudeau delivered speeches to the nation on a daily basis, complemented by frequent interventions by Doctor Theresa Tam, Canada's chief public health officer (Pathway 3, Step 1). Two general observations can be made based on my corpus of televised addresses. First, Trudeau's style of communication (Pathway 3, Step 2) broadly adhered to the principles of Crisis and Emergency Risk Communication recommended by the WHO and the CDC,^60^ in that it was primarily meant to convey concrete advice and health directives to the population based on the recommendations of the WHO and Public Health Canada, as well as to reassure citizens by expressing empathy and concern for their health and finances. Second, Trudeau's discourse aimed to justify the implementation of extraordinary economic measures (“the largest economic program in Canada's history”)^61^ and to lend strong moral support to the exceptional measures adopted by provincial governments and federal agencies, which included intrusive regulations such as stay-at-home orders (SAHOs) and quarantines for travelers (Pathway 3, Steps 3 and 4). This was done using a science-based language of “risk” that gradually evolved into a discourse of “threat” as the number of cases continued to rise across the nation (Pathway 3, Steps 2 and 4). Therefore, Trudeau's discourse generally aligned with Pathway 3, technocratic securitization with collaboration.

The expression “Team Canada,” an allusion to the country's national Olympic team, aptly captures the spirit of the prime minister's public addresses during the early onset of the pandemic. The catchphrase expressed the federal government's willingness to work collaboratively with an ensemble of other actors—national and provincial public health authorities, provincial premiers, the private sector, research institutions, the WHO, and foreign allies—to bolster a “whole-of-government” response “to keep Canadians safe.”^62^ “Addressing COVID-19 must be a Team Canada effort,” Trudeau stated on 13 March: “The provinces and territories are facing various levels of risks, but we will make sure that we align our response across the country.”^63^ The same logic of collaboration was seamlessly extended to national and international health authorities: “from the beginning, Canada's response has been based on the latest available science and advice from our world-class health professionals.”^64^ “[W]e can only overcome COVID-19 if we take action together as a global community…. That means making sure the World Health Organization^65^ and our public health agencies have the resources they need.”^66^

Trudeau's “Team Canada” approach to the pandemic was very much in keeping with the liberal internationalist foreign policy outlook of the Liberal Party of Canada (LPC),^67^ while also acknowledging legitimacy constraints imposed by its intended audience of Canadian citizens and provincial premiers. Whereas former Conservative prime minister Stephen Harper defended a neo-continentalist, realist, and anti-multilateralist approach to foreign policy and adopted a skeptical stance on scientific expertise and climate change, Trudeau won the 2015 election largely by embodying the opposite ideology.^68^ He also countered his predecessor's vision of an “open federalism” with strengthened provincial autonomy, turning instead to the LPC's traditional penchant for a strong federal state capable of implementing social programs in decentralized low politics domains such as the environment—a stance that again threw into sharp relief the centrifugal dynamics of Canadian federalism, and sparked staunch opposition from the federation's most autonomist provinces, especially Quebec and Alberta.^69^ Therefore, when Canada was hit by the first wave of the pandemic, Trudeau was disposed to display strong federal leadership anchored in a dual logic of 1) deference towards the policy advice of national and international health authorities and other experts, all of whom recommended decisive action and national coordination against the pandemic; and 2) recognition of the federal government's contested legitimacy to interfere in provincial governance by *imposing* country-wide health regulations.

On the one hand, partisan politics in Canada during the first phase of the crisis formed an ideal environment for scientific objectivation to produce a strong effect, which prevented the pandemic and public health measures from being politicized in any significant way by provincial premiers and federal party leaders. While radical right populism and conspiracist movements exist in Canada, these positions were marginalized in the federal party system of the time (that is, prior to the election of Pierre Poilievre as the head of the Conservative Party in September 2022). In 2019 and 2021, approximately 58 percent of the popular vote and 64 percent of the seats went to the LPC, the Bloc Québécois, and the New Democratic Party,^70^ all of which espouse liberal internationalism's deference towards multilateral institutions and global expertise. Indeed, the presence of “anti-vaxxers” among many of Conservative Members of Parliaments and partisans proved a significant source of embarrassment and an electoral liability for Conservative party leader Erin O’Toole in the 2021 election.^71^ Trudeau could therefore justify the exceptional measures adopted by all levels of government and urge his constituents to comply with public health directives by appealing to the public's “trust” in “doctors” and “the latest available science.”^72^

On the other hand, the federal government refrained from enacting the Emergency Management Act, which would have notably allowed it to bypass civil liberties and provincial powers to stage policy interventions and enforce lockdowns in the most affected cities and regions of the country. Provinces were left to determine for themselves if and when they issued measures falling within their constitutional purview such as SAHOs and indeed, there was a fair amount of variation in their respective public health strategies throughout the pandemic. In March 2020, however, all provinces were quick to implement partial or total lockdowns of their own volition, which serves as another testament to the power of scientific objectivation in Canada.^73^ Faced with an audience of provincial premiers who took COVID-19 seriously but were nonetheless wary of preserving their autonomy vis-à-vis the federal government,^74^ Trudeau responded by providing ample moral support to provincial decisions and reassuring Canadians that “all levels of governments are working together,” but without ever suggesting that the federal state was imposing common standards on the provinces.^75^

The success of exceptionalism in Canada indeed depended on Trudeau's ability to project an image of national unity and intergovernmental collaboration by avoiding a costly legitimacy contest with provincial premiers. Along with Trudeau's weekly phone calls with premiers, pre-existing intra-federal channels of communication allowed the provincial and federal governments to exchange information and resolve potential disagreements in a discreet manner, away from the public's eye, which helped keep intergovernmental conflicts to a minimum during the pandemic, in stark contrast with what happened in the US.^76^

Whereas in March, Trudeau's discourse focused on the “risks” COVID-19 posed to the most vulnerable Canadian citizens and strove to reassure the population that their government was “there for them” without drawing any explicit links between the measures taken against the virus and national security, a shift in rhetoric occurred at the beginning of April, when cases continued to rise vertiginously in Quebec and Ontario. Without abandoning the “Team Canada” trope, Trudeau reframed the pandemic as “the challenge of our generation,” and drew comparisons between citizens and their “grandparents,” who had shown their courage, solidarity, and sense of civic duty when they mobilized for the future of their country during the two World Wars. Canadians were once again being asked to “stand together” and “fight for every inch of ground against this disease” by complying with public health regulations in their respective provinces and, above all, *staying at home*.^77^ His rhetoric on SAHOs was in fact much more forceful than those of many premiers (“We all have a moral obligation to stay home”), as was his stance on travelers who failed to comply with quarantine regulations (“this kind of conduct [is] dangerous”).^78^

Overall, by repeatedly articulating the link between individual behaviour, individual health, and national security—together encompassing the welfare and future of the country in “unprecedented” times—Trudeau effectively deployed a securitizing discourse that conceived of the state's interests and security as inextricably enmeshed with those of the rest of the globe, and which cast the national economy and the health of individual Canadians as the referent objects of security. Meanwhile, the COVID-19 virus stood as the referent subject, in accordance with the dominant technocratic discourse linking human, national, and international security presented by the WHO: “Now more than ever, global cooperation is crucial not only to defeat this virus, but to address the great challenges of our time—challenges that transcend borders.”^79^ This discourse was deployed without recourse to any direct forms of power centralization or overt attempts to override provincial authority, although the adoption of an exceptionally robust package of economic measures to support individuals and businesses was considered by some premiers as a form of “soft” or “indirect” centralization, as it occasionally thwarted their own attempts to reopen parts of the economy by encouraging citizens to remain quarantined.^80^

In sum, because Trudeau's vision of sovereignty prior to the pandemic already involved notions of power delegation and subordination to a global “common good” and the protection of “human rights,” and because COVID-19 itself was not (yet) politicized in any significant manner in Canada, he was favourably disposed towards a technocratic and collaborative securitization of COVID-19, striking a compromise between respect for provincial jurisdictions and “soft” federal leadership and mainly taking the form of economic interventionism.

## “It's going to disappear.” Trump's politicization of COVID-19

In his 11 March 2020 speech to the nation, Trump depicted COVID-19 as an “enemy at the door” which could only be defeated by the “aggressive” action of the US federal government and the uniting of Americans behind their president;^81^ an unsurprising tone, considering that his entire presidency up to the onset of the pandemic had consisted of attempts to introduce sovereign decisionism in as many sectors of politics as possible.^82^ Indeed, in the weeks that followed the declaration of a national emergency on 13 March, Trump made several false statements that he alone possessed “full authority” to make decisions regarding the implementation of public health measures,^83^ in blatant disregard of constitutional limits on presidential power. However, his 13 April attempt to claim via tweet that it was “the decision of the President,” and not “the Governors [sic] decision to open the states,”^84^ was met by a unanimous rebuff from Republican and Democratic governors alike, showcasing the strength of American federalism and the clear limits that decentralized governance ultimately imposes on sovereign decisionism, even in times of emergency. While at first glance, Trump's discourse may appear consistent with Pathways 1 and 2 to exceptionalism, both of which involve securitization with power concentration, a sustained examination of Trump's rhetoric during the period eliminates both possibilities.

First, the initial impetus to securitize the pandemic came not from politicians and state officials, but from the CDC, which first flagged the virus on 17 January following the WHO's urgent calls to countries to increase preparations for testing and tracing;^85^ a sequence more consistent, a priori, with technocratic rather than classical securitization. Trump's response to technocratic pressure from the CDC through January to early March was to create the White House Coronavirus Task Force on 29 January. However, instead of framing COVID-19 as a threat necessitating robust measures, as outlined by CST, the president generally ignored expert advice and sought to keep the impeding pandemic *off* the governmental agenda, by multiplying unverified claims that the US government had the virus “under control,” that it would eventually “disappear,” and that it was no worse than “the common flu,” thus severely downplaying its virulence and high probability of super-spreading (Pathway 1, Step 1).^86^

Second, whether Trump's rhetoric can be labelled as “securitization” at all is disputable and deserves careful consideration (Pathway 1, Step 2).^87^ On the one hand, scientific objectivation and technocratic securitization of the virus, combined with rising rates of infection in the US in March 2020, ultimately compelled forty-three of the fifty American states to issue SAHOs between 19 March and 7 April and, in an exceptional manner, brought Republicans and Democrats together to pass a series of emergency bills providing financial assistance to quarantined businesses and individuals.^88^ On the other hand, most of these decisions were largely out of the president's hands, although Trump initially endorsed them to support his narrative that the American government was deploying the most “aggressive and comprehensive effort to confront a foreign virus in modern history.”^89^ These ringing endorsements of America's medical professionals later revealed themselves as a deliberate effort to *undermine* exceptionalism (Pathway 1, Step 2) by promising the audience a quick return to “normal politics,” a promise that contradicted even the most optimistic projections of epidemiologists at the time. Indeed, not only was the purported threat posed by COVID-19 downplayed rather than magnified, but co-optation of technocratic expertise remained limited and, in the end, amounted to a legitimacy contest between Trump and American public health officials over whom could advance the most credible claims concerning the evolution of the pandemic and the appropriateness of various measures and “treatments.”^90^ These systematic attempts at discrediting expertise (compounded with repeated refusals to mask and respect social distancing)^91^ ultimately culminated in Trump's threat to fire Anthony Fauci, the director of the National Institute of Allergy and Infectious Diseases, in November 2020.^92^ Trump pressured the CDC to conform its national pandemic guidelines to his political will, turning a blind eye to Republican governors who openly ignored the directives and criticizing Democratic leaders who chose to comply by maintaining their SAHOs, even going as far as to support the anti-lockdown protests erupting in many states via tweets such as “LIBERATE VIRGINIA.”^93^

Trump's discourse in the first months of the pandemic reflected the constraints imposed on him by his audience of state governors and American citizens, as well as his own conception of sovereignty which broadly derives from the ideological currents that historically underly understandings of legitimate political power within the GOP. First, in marked divergence from the Canadian context, partisan politics in the US have been characterized for the past decade by an intensifying combination of mass affective polarization and ideological polarization among state and congressional incumbents that has severely degraded the quality and consolidation of American democracy.^94^ Hence, the resulting “culture war” between Democrats and Republicans pitches a liberal and a conservative interpretation of American values and identity in a zero-sum contest wherein deference towards scientific and technocratic expertise on matters such as vaccination and climate change no longer constitute centrist positions capable of generating broad public adherence and an inter-partisan consensus in the federal party system and among sub-state leaders, as was still the case in Canada back in early 2020. Instead, either position is consistently embraced by only one side of two seemingly irreconcilable political camps that are respectively compelled to favour partisan competition over collaboration and compromise.^95^ By campaigning on the slogan “Make America Great Again” in 2016 and portraying himself as the only “strong man” who could defend the nation against the “destructive” forces of globalization and a “corrupt” political elite scheming to rob the country of its freedom through scientific “scams” and control of the “deep state,”^96^ Trump himself was a major contributor to these polarization dynamics.

Second, Trump's discourse on COVID-19 must also be contextualized by considering his beliefs regarding the proper functioning of American federalism. Underpinning Trump's response was the neo-liberal “anti-federalist” orientation that overtook the Republican party after the Reagan years; it seeks to preserve the states’ sovereignty against interference by the federal government, notably by advocating for drastic cuts in the funding of federal health and welfare agencies (starkly exemplified by his abolishment of Barack Obama's pandemic response office in 2018).^97^ Unlike in Canada, no formal or informal channels were mobilized to ensure regular contacts between the federated entities and the federal executive,^98^ forcing governors in need of federal assistance to clamour for the president's attention by making declarations in the media, directly contributing to the rapid politicization of the crisis. Most importantly, as noted by André Lecours et al., the vertical integration of the American party system provided Trump with considerable electoral incentives to publicly blame Democratic governors for “mismanaging” the pandemic and to engage in “transactional federalism” by rewarding state leaders who followed his personal wishes with federal resources.^99^ Variation in US state policies during the first year of the pandemic can largely be attributed to partisan cleavages: Democratic states adopted earlier and longer SAHOs on average than Republican states, and all seven states that never adopted SAHOs were Republican.^100^ Trump's discourse was therefore tailored specifically to the worldviews of his *chosen* audience of traditional supporters, right-leaning voters and Republican governors, and appears to have been motivated by a desire to improve his prospects for the November 2020 election by exploiting territorially entrenched polarization. Far from strengthening national sovereignty, his discourse weakened federal leadership (which hindered testing, tracing, and distribution of medical supplies by federal agencies across the country),^101^ reduced centralization (despite instrumental and widely rejected comments to the effect that the President held “absolute power” over the states), and continuously undermined the state of exceptionalism in the US.

On the other hand, Trump consistently supported one particular set of exceptional measures: border closures, travel restrictions, and restrictions on immigration.^102^ His 11 March speech and subsequent tweets grossly exaggerated the role of travelers and migrants in spreading the “Chinese virus” in the US,^103^ resulting in restrictions on travel from China, Iran, and Europe between 31 January and 14 March.^104^ Additional tweets casted blame on the WHO for its “incompetence” and “faulty” recommendation to maintain open borders, paving the way for Trump's decision to withdraw US funding, and then membership, from the organization, based on the claim that it was sold to Chinese interests.^105^ At first glance, Trump's hyperbolic depictions of the “threat” faced by the US imply that some form of securitizing discourse was indeed being deployed in favour of increased border controls and limitations on international mobility (Pathway 1, Step 2). However, and in contradiction with the WHO's technocratic human/national/international security discourse, Trump's discourse designated a *particular* understanding of American sovereignty (the one championed by the Republican side of the culture war) as the referent object of security, and alternatively framed the referent subject as either the virus itself *or* as a host of “enemies” (such as China, Democratic governors, and migrants) who were being held responsible for spreading it, failing to contain it, or using it as a false pretext to destroy the US from within by attacking the liberty of its people.

Therefore, what prevents Trump's overall discourse from qualifying as a properly “securitizing” speech are the many contradictions underlying it. By appealing to only one side of the electorate and portraying the subject referent in an inconsistent way—that is, by alternating between calling the virus a hoax and blaming America's “enemies” for worsening this “terrible” pandemic—he failed to objectify the purported threat by elevating it above normal politics. In effect, Trump's rhetoric maintained the pandemic *at the level of politicization*, that is, the *first step* of Pathway 1 to exceptionalism. While the case can certainly be made that he instrumentalized the pandemic to further securitize groups that had been previously targeted by his administration, these ongoing securitization processes should not be confused with the securitization of COVID-19 itself. Indeed, securitizing COVID-19 in the US context could not easily have been done *without* involuntarily reinforcing the authority of technocratic and sub-state actors, in a manner that would have been irreconcilable with Trump's own understanding of populist majoritarian sovereignty. This remains true despite growing scholarly concern that the US is undergoing a phase of democratic backsliding, as federal institutions and checks and balances in the country remain much stronger than in competitive-authoritarian regimes.^106^ These historical institutions partly explain why Trump ultimately lacked the necessary *legitimacy* to wrestle control of the pandemic issue from competing technocratic and sub-state authorities (although he did take back *some* control by withdrawing from the WHO). The other side of the equation is that, although already entrenched polarization in the US allowed Trump to counter some of the effects of scientific objectivation by politicizing the pandemic, it did not grant him the power and legitimacy to completely negate the influence of experts over the liberal-Democratic side of public opinion.

## Conclusion

This article's comparative analysis of Trudeau's and Trump's responses to the first wave of the COVID-19 pandemic demonstrates how considering the diverging conceptions of sovereignty espoused by heads of state and the legitimacy constraints with which they are faced can contribute to explaining whether and how they securitize “low politics” issues such as healthcare. Trudeau's televised public addresses evinced a technocratic and collaborative securitization of the pandemic that was consistent with his liberal internationalist views of state sovereignty and reflected the legitimacy constraints imposed on him by provincial autonomy in Canada. Meanwhile, Trump's speech to the nation and innumerable tweets persistently *undermined *exceptionalism in the US by exacerbating and playing into the intersection of federalism and partisan polarization, in an institutional and pre-electoral context in which securitizing the virus would have reinforced the authority of international and sub-state bodies in a manner incompatible with his own populist majoritarian understanding of sovereignty. Most significantly, neither Trump nor Trudeau used COVID-19 itself as a pretext to actively concentrate powers in the hands of the federal state. These findings, preliminary as they are due to the limited number of cases, nevertheless demonstrate that securitization is not *always* conducive to power centralization, that sovereignty and the securitization of low politics issues aren’t *necessarily* at odds, and that securitization does not *always* originate in the politicization of a particular issue but can instead result from scientific objectivation. Moreover, the Canadian case seems to suggest that high scientific objectivation and low levels of politicization within the domestic audience and political elites can create favorable conditions for the deployment of securitization discourses that do not overtly challenge the pre-existing institutional and political sharing of powers between central and sub-state authorities. The American case, for its part, shows that strong domestic politicization of (inter)national technocratic authority can heighten partisan polarization and provoke political legitimacy contests between orders of government. Further research should broaden the empirical investigation to other federal, as well as non-federal and unitary, states, to better understand how polarization, politicization of expertise and global authority, and understandings of internal and external sovereignty shape the ideational context surrounding political decision-making and leaders’ legitimacy constraints in the securitization of low politics. Indeed, although the analytical framework presented in this article was first developed to analyze securitization processes in federal states, it can also be applied to the study of low politics securitization in any state with a multi-level diffusion of power to substate and local authorities.

This article also aimed to open a conversation about the unfolding of securitization processes over time and to further ongoing discussions about their consequences for democracy. An emerging branch of scholarship in the global health regime literature suggests that exceptionalism may arise in times of emergency based on a logic of “risk” as opposed to a rhetoric of “threat,” the former being more compatible with the preservation of civil liberties.^107^ However, the underlying paradox of the global health regime, which exacerbates the tensions between deliberative democratic decentralization and centralized rule as well as between political deliberation and scientific objectivation by encouraging an alignment between human, national, and international security, has not been sufficiently addressed. Nevertheless, while sovereign decisionism may be undesirable in low politics domains, inaction and complacency in the face of increasing hazards to human welfare in the Anthropocene are equally concerning: as showcased by Joe Biden's failure to counter the escalation of the pandemic in the USA, followed by the rapid *de*securitization of the virus in Canada and other countries after the first vaccination campaigns, short-term handling of threats does not easily translate to long-term management of risks.^108^ Consequently, the jury's still out on what kinds of *long-term* solutions will be devised to meet coming environmental and health challenges to humanity, and whether these solutions will involve greater *diffusion*, or *concentration*, of state sovereignty—a question most relevant for students of federalism and multi-level governance as well as specialists of international security.

## Notes

1. Amber Phillips, “President Trump's address to the nation on coronavirus, annotated,” *The Washington Post*, 11 March 2020, https://www.washingtonpost.com/politics/2020/03/11/trump-coronavirus-address-transcript/ (accessed 15 October 2021).

2. “Prime Minister's remarks on Canada's response to COVID-19,” Prime Minister of Canada Justin Trudeau, 10 March 2020, https://pm.gc.ca/en/videos/2020/03/11/prime-ministers-remarks-canadas-response-COVID-19 (accessed 15 October 2021).

3. Carl Schmitt, *Political Theology: Four Chapters on the Concept of Sovereignty* (Chicago: Chicago University Press, 2005 [1922]), 7.

4. Tine Hanrieder and Christian Kreuder-Sonnen, “WHO decides on the exception? Securitization and emergency governance in global health,” *Security Dialogue* 45, no. 4 (2014): 331–348.

5. Craig Albert, Amado Baez, and Joshua Rutland, “Human security as biosecurity. Reconceptualizing national security threats in the time of COVID-19,” *Politics and the Life Sciences* 40, no. 1 (Spring 2021): 86–88.

6. Clare Wenham, Joshua W. Busby, Jeremy Youde, and Asha Herten-Crabb, “From imperialism to the ‘Golden Age’ to the Great Lockdown: The politics of global health governance,” *Annual Review of Political Science* 26 (2023): 431–450.

7. Melissa Curley and Jonathan Herington, “The securitisation of avian influenza: International discourses and domestic politics in Asia,” *Review of International Studies* 37, no. 1 (2011): 165.

8. Shirley V. Scott, “The securitization of climate change in world politics: How close have we come and would full securitization enhance the efficacy of global climate change policy?” *Review of European Community & International Environmental Law* 21, no. 3 (2012): 220–230.

9. See, for example: Erik Brattberg and Mark Rhinard, “Multilevel governance and complex threats: The case of pandemic preparedness in the European Union and the United States,” *Global Health Governance* 5, no. 1 (2011): 1–21; Mely Caballero-Anthony, *Negotiating Governance on Non-Traditional Security in Southeast Asia and Beyond* (New York: Columbia University Press, 2018).

10. Daniel J. Elazar, “Contrasting unitary and federal systems.” *International Political Science Review* 18, no. 3 (1997): 237–251.

11. See, for example: Nico Steytler, ed., *Comparative Federalism and COVID-19. Combating the Pandemic* (London: Routledge, 2022); Jan Rovny et al., “Contesting Covid: The ideological bases of partisan responses to the Covid-19 pandemic,” *European Journal of Political Research* 61 (2022): 1155–1164.

12. See, for example: Johanna Schnabel and Yvonne Hegele, “Explaining intergovernmental coordination during the COVID-19 pandemic: Responses in Australia, Canada, Germany, and Switzerland,” *Publius: The Journal of Federalism* 51, no. 4 (2021): 537–569.

13. Thierry Balzacq, “The three facts of securitization: Political agency, audience and context,” *European Journal of International Relations* 11, no. 2 (2005): 181–185.

14. Grasping all dimensions of political decision-making in times of crisis is a colossal task requiring the consideration of multiple variables, including, for instance, a leader's personality or prior experience of a state administration with a similar crisis event (for an exhaustive list of these potential variables, see Arjen Boin, Allan McConnell, and Paul ’t Hart, *Governing the Pandemic: The Politics of Navigating a Mega-Crisis* [Cham: Palgrave Macmillan, 2021]). This article's main objective, however, is not to provide a comprehensive analysis of Trump and Trudeau's crisis management and communication strategies, nor is it primarily focused on the *individual* level of analysis: instead, by considering both leaders as *pragmatic agents*, it seeks to resituate their agency—and that of the federal executive, in Trudeau's case—within a broader structural context related to contending contemporary conceptions of sovereignty. For studies on political leadership and crisis communication in federal states during the pandemic, see, for example, Marianne Kneuer and Stefan Wallaschek, “Framing COVID-19: Public leadership and crisis communication by Chancellor Angela Merkel during the pandemic in 2020,” *German Politics* 32, no. 4 (2023): 686–709. See also Johanna Schnabel, Paul Anderson, and Fabrizio De Francesco, “Multilevel governance and political leadership: Crisis communication in Germany, Italy, and the United Kingdom during the COVID-19 pandemic,” *Journal of European Public Policy,* 28 April 2023, https://doi.org/10.1080/13501763.2023.2199784.

15. Barry Buzan, “Rethinking security after the Cold War,” *Cooperation and Conflict* 31, no. 2 (1997): 9–12.

16. Ibid., 13–14.; Ole Waever, “Securitization and desecuritization,” in Ronnie D. Lipschutz, ed., *On Security* (New York: Columbia University Press, 1995), 46–86.

17. Barry Buzan and Lene Hansen, “The key questions in International Security Studies: The state, politics and epistemology,” in Barry Buzan and Lene Hansen, eds., *The Evolution of International Security Studies* (Cambridge: Cambridge University Press, 2009), 21–28.

18. Thierry Balzacq, Sarah Léonard, and Jan Ruzicka, “'Securitization revisited: Theory and cases,” *International Relations* 30, no. 4 (2016): 494–531.

19. Buzan and Hansen, “The key questions in ISS,” 34.

20. For a comprehensive study of governmental responses to COVID-19 utilizing this framework, see Boin et al., *Governing the Pandemic*, 19–37.

21. Ibid., 20–21.

22. Ibid., 19–37 and 43–60.

23. Balzacq, Léonard, and Ruzicka, “Securitization revisited,” 499–500.

24. Juha. A. Vuori, “Constructivism and Securitization Studies,” in Myriam Dunn Cavelty and Thierry Balzacq, eds., *Routledge Handbook of Security Studies* (London: Routledge, 2016), 65.

25. Balzacq, “The three facts,” 181–185.

26. Lene Hansen, *Security as Practice: Discourse Analysis and the Bosnian War* (New York: Routledge, 2005).

27. Constructivists in IR such as Ted Hopf and Alexander Wend typically refer to conceptions of sovereignty through the concept of “national identity.” On the relationship between national identity and foreign policy, see Peter Katzenstein, ed., *The Culture of National Security: Norms and Identity in World Politics* (New York: Columbia University Press, 1996). Party ideology may also be used to indirectly refer to understandings of sovereignty and has been shown to have had a great influence on party and governmental responses to the pandemic in Europe, but the notion of “conceptions of sovereignty” was retained for this study to broaden the scope of analysis beyond the dynamics of electoral competition. See, for instance, Rovny et al., “Contesting Covid,” 1155–1164.

28. Vuori, “Constructivism and Securitization Studies,” 66.

29. Liesbet Hooghe and Gary Marks, “Unravelling the central state, but how? Types of multi-level governance,” *American Political Science Review* 97, no. 2 (2003): 233.

30. Ibid., 236.

31. Daniel Elazar, *The Ends of Federalism: Notes Towards a Theory of Federal Political Arrangements* (Philadelphia: Temple University, 1976), 36–37.

32. Johanne Poirier, Cheryl Saunders, and John Kincaid, eds., *Intergovernmental Relations in Federal Systems* (Oxford: Oxford University Press, 2015), 4.

33. Richard Simeon and Beryl A. Radin, “Federalism,” in Paul J. Quirk, ed., *The United States and Canada: How Two Democracies Differ and Why It Matters* (Oxford: Oxford University Press, 2019), 154–155.

34. Yvonne Hegele and Johanna Schnabel, “Federalism and the management of the COVID-19 crisis: Centralisation, decentralisation and (non-)coordination,” *West European Politics* 44, no. 5–6 (2021): 1052–1076.

35. Kenneth Wheare, *Federal Government* (Oxford: Oxford University Press, 1967), 168.

36. Curley and Herington, “The securitization of avian influenza,” 158–159.

37. Hooghe and Marks, “Unravelling the central state,” 236.

38. Sarah Davies, “Securitizing infectious disease,” *International Affairs* 84, no. 2 (March 2008): 301.

39. Stephan Elbe, “Should HIV/AIDS be securitized? The ethical dilemmas of linking HIV/AIDS and security,” *International Studies Quaterly* 50, no. 1 (2006): 119–144.

40. Albert, Baez, and Rutland, “Human security,” 86–88; Buzan and Hansen, “The key questions in ISS,” 36.

41. Michael Zürn, *A Theory of Global Governance* (Oxford: Oxford University Press, 2018), 4.

42. Anders Esmark, “How does crisis affect the conflict between technocracy and populism? Lessons from the COVID-19 pandemic,” *Politics* 43, no. 4 (2021): 10, https://doi.org/10.1177/02633957211049965.

43. Catherine E. De Vries, Sarah B. Hobolt, and Stephanie Walter, “Politicizing international cooperation: The mass public, political entrepreneurs, and political opportunity structures,” *International Organization* 75 (Spring 2021): 306–332.

44. Liesbet Hooghe and Gary Marks, “Cleavage theory meets Europe's crises: Lipset, Rokkan, and the transnational cleavage,” *Journal of European Public Policy* 25, no.1 (2018): 109–135.

45. Steven Webster and Alan Abramowitz, “The ideological foundations of affective polarization in the U.S. electorate,” *American Politics Research* 45, no. 4 (2017): 621–647; Paul Pierson and Eric Schickler, “Madison's constitution under stress: A developmental analysis of political polarization,” *Annual Review of Political Science* 23 (2020): 37–58.

46. James N. Druckman et al., “How affective polarization shapes Americans’ political beliefs: A study of response to the COVID-19 pandemic,” *Journal of Experimental Political Science* 8, no. 3 (2021): 223–234.

47. Esmark, “How does crisis,” 3.

48. Trine Villumsen Berling, “Science and securitization: Objectivation, the authority of the speaker and mobilization of scientific facts,” *Security Dialogue* 42, no. 4–5 (2011): 385–386.

49. Esmark, “How does crisis,” 6.

50. University of Oxford, “COVID-19: Stringency index,” Our World in Data, https://ourworldindata.org/covid-stringency-index (accessed 22 September 2021).

51. Esmark, “How does crisis,” 8–9.

52. Throughout the pandemic, the WHO pushed for a “whole-of-government” and “whole-of-society” approach, striking “a fine balance between protecting health, minimizing economic and social disruption, and respecting human rights.” Member states were furthermore urged to “proceed with caution” before lifting lockdowns and other public health and social measures (PHSM) to avoid compromising long-term recovery. See Tedros Adhanom Ghebreyesus, “WHO Director-General's opening remarks at the World Health Assembly,” World Health Organization, https://www.who.int/director-general/speeches/detail/who-director-general-s-opening-remarks-at-the-world-health-assembly (accessed 2 November 2021); Christian Kaunert, Sarah Leonard, and Ori Wertman, “Securitization of COVID-19 as a security norm: WHO norm entrepreneurship and norm cascading,” *Social Sciences* 11, no. 266 (2022): 1–19.

53. Jessica Kirk and Matt McDonald, “The politics of exceptionalism: Securitization and COVID-19,” *Global Studies Quarterly* 1 (2021): 4.

54. Esmark, “How does crisis,” 2.

55. Kirk and McDonald, “The politics of exceptionalism,” 1–12.

56. Jessica Kirk, “From threat to risk? Exceptionalism and the logics of health security,” *International Studies Quarterly* 64, no. 2 (2020): 266–276.

57. University of Oxford, “COVID-19: Cases,” Our World in Data, https://ourworldindata.org/covid-cases (accessed 13 November 2022).

58. Frédéric Boily, “Justin Trudeau, les premiers ministres provinciaux et la crise sanitaire : leadership et paternalisme doux,” *Études canadiennes / Canadian Studies*, no. 89 (2020): 339.

59. Hélène Buzzetti and Marie Vastel, “Le Canada ferme ses frontières,” *Le Devoir*, 17 March 2020, https://www.ledevoir.com/societe/sante/575058/trudeau-annonce-de-nouvelles-mesures-dans-la-lutte-contre-la-COVID-19 (accessed 2 November 2021).

60. “Emergencies: Risk communication,” World Health Organization, https://www.who.int/news-room/questions-and-answers/item/emergencies-risk-communication (accessed 13 November 2021).

61. “Updating Canadians on the rollout of the Canada Emergency Response Benefit,” Prime Minister of Canada Justin Trudeau, 1 April 2020, https://pm.gc.ca/en/videos/2020/04/01/updating-canadians-rollout-canada-emergency-response-benefit (accessed 15 October 2021).

62. “Prime Minister's remarks on Canada's response to COVID-19,” Prime Minister of Canada Justin Trudeau, 10 March 2020, https://pm.gc.ca/en/videos/2020/03/11/prime-ministers-remarks-canadas-response-COVID-19 (accessed 15 October 2021).

63. “Prime Minister Trudeau updates Canadians on COVID-19,” Prime Minister of Canada Justin Trudeau, 12 March 2020, https://pm.gc.ca/en/videos/2020/03/13/prime-minister-trudeau-updates-canadians-COVID-19 (accessed 16 October 2021).

64. “The Government of Canada's response to COVID-19,” Prime Minister of Canada Justin Trudeau, 16 March 2020, https://pm.gc.ca/en/videos/2020/03/16/government-canadas-response-COVID-19 (accessed 16 October 2021).

65. This did not prevent Trudeau from later recognizing, in reaction to Trump's decision to withdraw American funding from the WHO, that the organization would have to respond to member states’ concerns about its relationship with China. He nonetheless reiterated Canada's unconditional support towards multilateral organizations and the WHO's authority in the health domain. Rachel Gilmore, “‘No institution is perfect’: Trudeau promises to support WHO in wake of Trump ultimatum,” CTV News, 19 May 2020, https://www.ctvnews.ca/politics/no-institution-is-perfect-trudeau-promises-to-support-who-in-wake-of-trump-ultimatum-1.4944875 (accessed 2 November 2021).

66. “Canada's plan to support farmers and mobilize science to fight COVID-19,” Prime Minister of Canada Justin Trudeau, 23 March 2020, https://pm.gc.ca/en/videos/2020/03/23/canadas-plan-support-farmers-and-mobilize-science-fight-COVID-19 (accessed on 16 October 2021).

67. Norman Hillman and Philippe Lagassé, eds., *Justin Trudeau and Canadian Foreign Policy* (Basingstoke: Palgrave MacMillan, 2018), 1–16.

68. Brooke Jeffrey, “Canadian liberalism as a distinct political tradition,” in David McGrane and Neil Hibbert, eds., *Applied Political Theory and Canadian Politics* (Toronto: University of Toronto Press, 2019), 62–66.

69. Mark Winfield and Douglas Macdonald, “Federalism and Canadian climate change policy,” in Herman Bakvis and Grace Skogstad, eds., *Canadian Federalism: Performance, Effectiveness, and Legitimacy* (Toronto: University of Toronto Press, 2020), 365.

70. “Elections Canada,” Government of Canada, https://www.elections.ca/home.aspx (accessed 21 November 2021).

71. Stephanie Taylor, “Erin O’Toole criticizes comments by Tory MPs on COVID-19 vaccines: ‘Not appropriate,’” Global News, 8 November 2021, https://globalnews.ca/news/8358836/conservative-mps-covid-vaccine-comments-otoole/ (accessed 13 November 2021).

72. “The Government of Canada's response to COVID-19.”

73. Indeed, even the United Conservative Party of Alberta leader Jason Kenney, the premier who was the most reluctant to implement health measures, responded to the first wave by declaring a state of emergency and instituting a lockdown which lasted until 15 May; a SAHO was issued in December 2020 when rates of infection began to spike. See Emily Cameron-Blake et al., “Variation in the Canadian provincial and territorial responses to COVID-19,” BSG-WP-2021/039 (BSG Working Paper Series, University of Oxford and Blavatnik School of Government, March 2021): 4, 11, 15.

74. André Lecours, Daniel Béland, Alan Fenna, Tracy Beck Fenwick, and Mireille Paquet, “Explaining intergovernmental conflict in the COVID-19 crisis: The United States, Canada, and Australia,” *Publius: The Journal of Federalism* 51, no. 4 (2021): 523.

75. “Prime Minister Trudeau updates Canadians on COVID-19.”

76. Lecours et al., “Explaining,” 523–525.

77. “The COVID-19 pandemic is the challenge of our generation,” Prime Minister of Canada Justin Trudeau, 12 April 2020, https://pm.gc.ca/en/videos/2020/04/12/COVID-19-pandemic-challenge-our-generation (accessed 16 October 2021).

78. “Updating Canadians on the COVID-19 situation,” Prime Minister of Canada Justin Trudeau, 26 March 2020, https://pm.gc.ca/en/videos/2020/03/26/updating-canadians-COVID-19-situation (accessed on 16 October 2021). In another televised address given on 12 June, the prime minister made his case for the maintenance of quarantine measures even more explicit: “In country after country…a pattern is emerging. People make sacrifices needed to flatten the curve, but then, when governments decide to restart the economy, cases begin to rise again. …Canada must learn from these lessons. It's clear that we have to make safety and control of this virus a top priority in this restart.” See “Announcing new screening measures for air passengers,” Prime Minister of Canada Justin Trudeau, 12 June 2020, https://pm.gc.ca/en/videos/2020/06/12/announcing-new-screening-measures-air-passengers (accessed 15 October 2021).

79. “Remarks on UN Security Council and the new mobile app to help notify Canadians of COVID-19 exposure,” Prime Minister of Canada Justin Trudeau, 18 June 2020, https://pm.gc.ca/en/videos/2020/06/18/remarks-security-council-and-new-mobile-app-help-notify-canadians-COVID-19 (accessed on 15 October 2021).

80. Boily, “Justin Trudeau, les premiers ministres provinciaux et la crise sanitaire,” 340–341; Lecours et al., “Explaining,” 525.

81. Phillips, “President Trump's address.”

82. Robert Kaufman and Stephan Haggard, “Democratic decline in the United States: What can we learn from middle-income backsliding?” *Perspectives on Politics* 17, no. 2 (2019): 424–427.

83. Christian Paz, “All the President's lies about the coronavirus,” *The Atlantic*, 2 November 2020, https://www.theatlantic.com/politics/archive/2020/11/trumps-lies-about-coronavirus/608647/ (accessed 13 November 2021).

84. Elizabeth Thomas and Jordyn Phelps, “Trump claims he, not governors, has power over states on deciding reopening country,” ABC News, 13 April 2020, https://abcnews.go.com/Politics/trump-claims-governors-power-states-deciding-reopening-country/story?id=70119115 (accessed 13 November 2021).

85. Paul Rutledge, “Trump, COVID-19, and the war on expertise,” *American Review of Public Administration* 50, no. 6–7 (2020): 506.

86. Daniel Wolf and Daniel Dale, “'It's going to disappear': A timeline of Trump's claims that COVID-19 will vanish,” *CNN News*, 31 October 2020, https://www.cnn.com/interactive/2020/10/politics/covid-disappearing-trump-comment-tracker/ (accessed 14 November 2021); See also Rutledge, “Trump, COVID-19,” 506–507.

87. Evidence for the securitization of COVID-19 by Donald Trump provided by the extant literature is at best mixed, with a majority of scholars agreeing that he downplayed the virus's severity far more than he magnified its threatening status. See, for instance, Jessica Kirk, “’The cure cannot be worse than the problem’: Securitising the securitisation of COVID-19 in the USA,” *Contemporary Politics* 29, no. 2 (2022): 141–160, https://doi.org/10.1080/13569775.2022.2095762 (accessed 2 September 2022).

88. John Kincaid and Wesley Leckrone, “Partisan fractures in US federalism's COVID-19 policy responses,” *State and Local Government Review* 52, no. 4 (2020): 300.

89. Phillips, “President Trump's address.”

90. Rutledge, “Trump, COVID-19,” 506–508.

91. Gideon Lasco, “Medical populism and the COVID-19 pandemic,” *Global Public Health* 15, no. 10 (2020): 1422–1423.

92. Paulina Villegas, “Trump says he might fire Fauci. Technically, he can’t,” *The Washington Post*, 2 November 2020, https://www.washingtonpost.com/health/2020/11/02/can-trump-fire-fauci/ (accessed on 13 November 2021).

93. Kincaid and Leckrone, “Partisan fractures,” 303.

94. Pierson and Schickler, “Madison's constitution”; Steven Levitsky and Daniel Ziblatt, *How Democracies Die* (New York: Crown Publishing Group, 2018).

95. Ibid.

96. Mike McIntire, Kate Yourish, and Larry Buchanan, “In Trump's Twitter feed: Conspiracy-mongers, racists and spies,” *The New York Times*, 2 November 2019, https://www.nytimes.com/interactive/2019/11/02/us/politics/trump-twitter-disinformation.html (accessed 12 November 2021).

97. John Agnew, “American ‘populism’ and the spatial contradictions of US government in the time of COVID-19,” *Geopolítica(s)* 11, no. Especial (2020): 20–22.

98. Lecours et al., “Explaining,” 519–520.

99. Ibid., 519.

100. Kincaid and Leckrone, “Partisan fractures,” 302.

101. Ibid., 299.

102. Agnew, “American populism,” 22.

103. Paz, “All the President's lies.”

104. Philip Wallach and Justus Myers, “The Federal government's coronavirus response—public health timeline,” Brookings, 31 March 2020, https://www.brookings.edu/research/the-federal-governments-coronavirus-actions-and-failures-timeline-and-themes/ (accessed 15 October 2021).

105. For example, Donald Trump (@realdonaldtrump), “The W.H.O. really blew it. For some reason, funded largely by the United States, yet very China centric. We will be giving that a good look. Fortunately I rejected their advice on keeping our borders open to China early on. Why did they give us such a faulty recommendation?” Twitter, 7 April 2020, https://twitter.com/realDonaldTrump/status/124754070129163878 (accessed 15 October 2021); Paz, “All the President's lies.”

106. Kaufman and Haggard, “Democratic decline,” 425–427.

107. Kirk and McDonald, “The politics of exceptionalism,” 4.

108. This normalization of the pandemic occurred despite the WHO's repeated reminders that Covid “remains an acute global emergency.” See World Health Organization, “COVID-19 policy briefs,” World Health Organization, https://www.who.int/emergencies/diseases/novel-coronavirus-2019/COVID-19-policy-briefs (accessed 28 February 2023); Jamey Keaten, “COVID-19 restrictions loosen around the world amid hopes Omicron has peaked,” Global News, The Associated Press, 2 February 2022, https://globalnews.ca/news/8591232/COVID-19-restrictions-loosen-world/ (accessed 28 February 2023).

